# How decoy options ferment choice biases in real-world consumer decision-making

**DOI:** 10.1038/s41539-025-00341-2

**Published:** 2025-08-22

**Authors:** Sean Devine, James Goulding, John Harvey, Anya Skatova, A. Ross Otto

**Affiliations:** 1 Department of Psychology, McGill University, Montreal, Canada; 2 N/LAB, Nottingham University Business School, Nottingham, UK; 3 Digital Footprints Lab & Medical Research Council Integrative Epidemiology Unit at the University of Bristol, Population Health Science, Bristol Medical School, University of Bristol, Bristol, UK

**Keywords:** Human behaviour, Decision making

## Abstract

The decoy effect describes a bias in which people’s choices between two valuable options are swayed by a third, inferior, “decoy” option. Despite being documented in lab settings, relatively little work has investigated whether decoy effects occur “in the wild” where consumers face large, diverse choice sets. We employ a new methodology to examine the impact of decoy options on purchase decisions using a dataset of 3.6 million UK grocery-store wine transactions. Results indicate that when comparing wines that vary in quality and price across contexts, the presence of dominated (i.e., inferior) decoy options increased consumers’ likelihood of choosing a target option—a hallmark of the well-documented attraction effect. The strength of these effects was modest overall (roughly 1% change in preference) and, interestingly, depended on consumers’ idiosyncratic histories of experience. Our study provides a proof of principle demonstrating that these sorts of context effects are detectable in richer, complex real-world consumer choice settings.

## Introduction

Central to theories of decision-making is the notion that human information processing is limited in capacity^1^. One classic manifestation of these constraints, when faced with the demands of a complex decision environment, is the context-dependent nature of human preference: our preference for an option depends not only that option’s own intrinsic value, but also the values of other, often irrelevant options^2–4^. Consider for example the choice a shopper faces between two wines, where the value of an option is computed across two dimensions: quality and price. When deliberating about which wine to choose among more than two desirable but competing options, a key tenet of rational (economic) choice—the “independence of irrelevant alternatives”—prescribes that decision-makers should ignore irrelevant, inferior alternative options^5,6^. According to this axiom, a shopper’s propensity to choose between two otherwise equally preferred wines should be not influenced by the introduction of a third option that is objectively inferior to one of the focal options. However, a large body of work suggests that both people^7–10^ and animals^11,12^ routinely violate this axiom in their decisions, finding that the introduction of irrelevant “decoy” options into a choice set systematically biases decision-making^3^.

Research examining decoy effects date back several decades, with early studies conducted by Huber and colleagues^7^ and Tversky and Simonson^4^ demonstrating that individuals’ relative preference between two different options could be, under specific circumstances, biased by the introduction of a third less attractive option. Since these initial studies, a spate of work has investigated the cognitive mechanisms underpinning decoy effects in consumer choice^3,13–19^. Despite these theoretical advancements, the specific conditions under which decoy effects take hold remain the subject of debate^20,21^. For example, concerted replication attempts by Frederick et al. and Yang and Lynn found that decoy effects could only be observed in stylized laboratory settings using numeric (as opposed to visual) representations of options’ attribute values^22,23^ (see also ref. ^24^ for an attempt to replicate these results in a field experiment).

These heterogenous results highlight how the observability of such decoy depends on, among other things, the configuration of options, attribute types, and product categories constituting the choice set in question^21^. While well-controlled laboratory studies have characterized the phenomenology of decoy effects and have informed our computational understanding of context effects^15,18,25,26^, relatively little work has investigated the extent to which decoy effects actually occur “in the wild” in larger option sets, as exemplified in real-world consumer choice. Previous studies have been limited in finding real-world evidence or these sorts of decoy effects manifest in shoppers’ choices, because, for example, the products under consideration were not amenable to quantifying option quality^27^, or required aggregation across consumer choices, obscuring potentially interesting features of both the consumers and the choice environment that may drive preference^28^. With respect to the latter point, unlike in tightly constrained laboratory settings, the choice sets facing real-world decision-makers are often arbitrarily large (e.g., a choice of 20–30 different wines in a convenience store), composed of options whose attribute values may not be readily observable (e.g., the quality of wine), and informed by decision-makers’ idiosyncratic past experiences with the options.

Understanding if, and how such decoy effects play out in consumers’ real-world purchasing decisions is of considerable practical and theoretical importance. Practically, even small decoy effects in real-world consumer choice can hold sizable economic consequences both for consumers and businesses—a 1% change in choice behavior, in the aggregate, can lead to hundreds of thousands of dollars in profits or losses^28^. Theoretically, interrogating these effects “in the wild” is not only important for understanding the boundary conditions of these well-studied laboratory effects (e.g.,^29–31^), but also to examine if other moderating variables that exist in complex real-world decision environments allow them to be expressed at all^22^. This latter point is particularly important, given recent proposals in the literature that the true magnitude of decoy effects may be overstated by the artificial, albeit well-controlled, nature of tasks employed in laboratory choice studies^22,23,32–34^ in contrast to naturalistic consumer choice, where multiple sources of bias may influence individuals’ preferences (e.g., anchoring effects;^35^). Moreover, experimental work on decoy effects has focused almost exclusively on trinary choice paradigms, in which the preference between two target options is tested in the presence (versus absence) of a single decoy option^22,23^. Real-world consumer choices settings, however, often entail a choice between several options, with the possibility that multiple, inferior decoys are present and could systematically bias choices between (superior) target options. An open question, therefore, concerns how clusters of decoy options influences real-world choice, beyond contrived trinary choice situations.

**Fig. 1:**
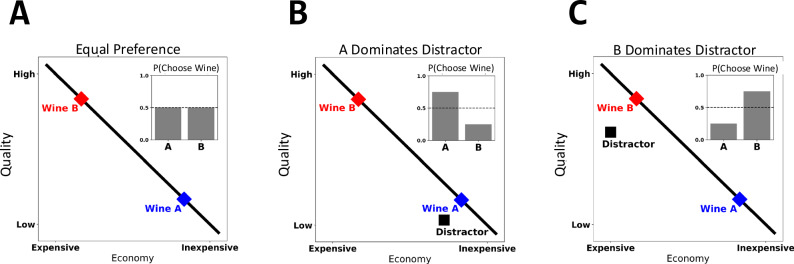
Toy illustration of the attraction effect. Illustration of the canonical attraction effect with a single distractor. **A** When no distractor options are present, individuals are indifferent between Wine A and Wine B. **B** When Wine A dominates the distractor or ‘decoy’ option, preference shifts towards Wine A. **C** When Wine B dominates the distractor or ‘decoy’ option, preference shifts towards Wine B.

In the present study we introduce a novel approach to probe for a canonical decoy effect in large choice sets— termed the *attraction effect*—in a massive consumer purchasing dataset. To illustrate the attraction effect, consider a scenario in which a consumer considers two wines: a high quality, but expensive wine (Wine A) and an inexpensive, but low-quality wine (Wine B; see Fig. 1A). Critically, the attributes defining these two options—price and quality—trade off, such that neither wine is superior to the other wine in both attributes. This results in indifference between the two options: on average, consumers should choose Wine A 50% of the time and Wine B 50% of the time (Fig. 1A). The attraction effect occurs if a third, ‘distractor’ option is introduced into the choice set which is similar, but, inferior to one of the focal options. Specifically, the attraction effect takes hold when the distractor option is ‘dominated’ by one (but not both) of the target options—that is, Wine A is superior in both price and quality than the distractor option, but Wine B is superior in only one dimension (quality; see Fig. 1B). The attraction effect is characterized by an increased preference for Wine A over Wine B, despite the fact that decision-makers have equal preference for the two target options in the absence in the absence of distractor items^3,7,15^. An equivalent, but opposite, attraction effect also obtains when the distractor option is dominated by Wine B, but not Wine A, resulting in stronger preference for Wine B (Fig. 1C).

Here we investigate if the phenomenon of decoy effects occurs in real-world consumer purchasing decisions, where individuals encounter a multitude of choices rather than a single decoy option. We analyze 3.6 M wine purchases in a retail dataset from a large grocery chain in the United Kingdom. We opted to examine wine choice as an instantiation of multi-attribute decision-making because it has a number of desirable properties for the present analysis. First, consumer psychology researchers have extensively examined wine purchasing behavior and have developed rich characterizations of decision variables, preferences, and motivations^36,37^. In particular, this body of work highlights the combined influence of internal (e.g., consumption history) and external (e.g., price, perceived quality) sources of information upon wine purchasing decisions^38–40^. This complex, multi-attribute decision problem makes wine an ideal product category for examining decoy effects in real-world consumer choice. In particular, wines vary considerably on two important, quantifiable attributes—price and quality—which demonstrably (1) influence consumers’ purchase decisions, and (2) trade off with one another^41^, mirroring the design of traditional laboratory decoy effect experiments^3,26^. Second, wine bottles are typically sold in a standard size (unlike other categories such as snack foods or bottled water), which means that product size should not be a relevant attribute in choices between wines. Third, in our dataset, we observed that most consumers purchased only a single bottle wine during a store visit, which suggests that the available wine options effectively compete against one another.

In this massive purchase dataset, we examine whether choices between pairs of target options—operationalized as popular wines that trade off in price and quality but are equally preferred across individuals—are influenced by varying contexts in which they appear. Because these ubiquitous target options appeared within diverse choice sets at different times, owing to variability in available wines across store locations, we rigorously test whether preferences between target pairs are systematically influenced by the presence of dominated distractor options. If so, this would suggest that the well-characterized attraction effect not only manifests in real-world decision-making contexts, but does so even in large choice sets and despite other environmental (e.g., time of day) and psychological variables (e.g., individual differences) moderating purchase decisions.

Leveraging the scale and heterogeneity of these choice contexts—and extending beyond traditional laboratory trinary choice paradigms—which are typically comprised of a single distractor option^3,8^—we investigate how the distribution of (multiple) distractor options influences preference between target options, With this approach, we can also examine how the number of distractor options dominated by a target item impacts the magnitude of the observed attraction effect. Finally, we investigate the extent to which differences in consumers’ purchasing histories modulate the strength of the attraction effect, suggesting boundary conditions for these observed decoy effects in naturalistic settings^22,23^.

## Methods

Here we present our method to analyze decoy effects in large scale, real-world purchasing datasets, describing in detail the data sources and statistical procedures used. Readers seeking an intuitive understanding of the dataset and analyses are encouraged to advance to the Results section.

### Dataset

**Fig. 2:**
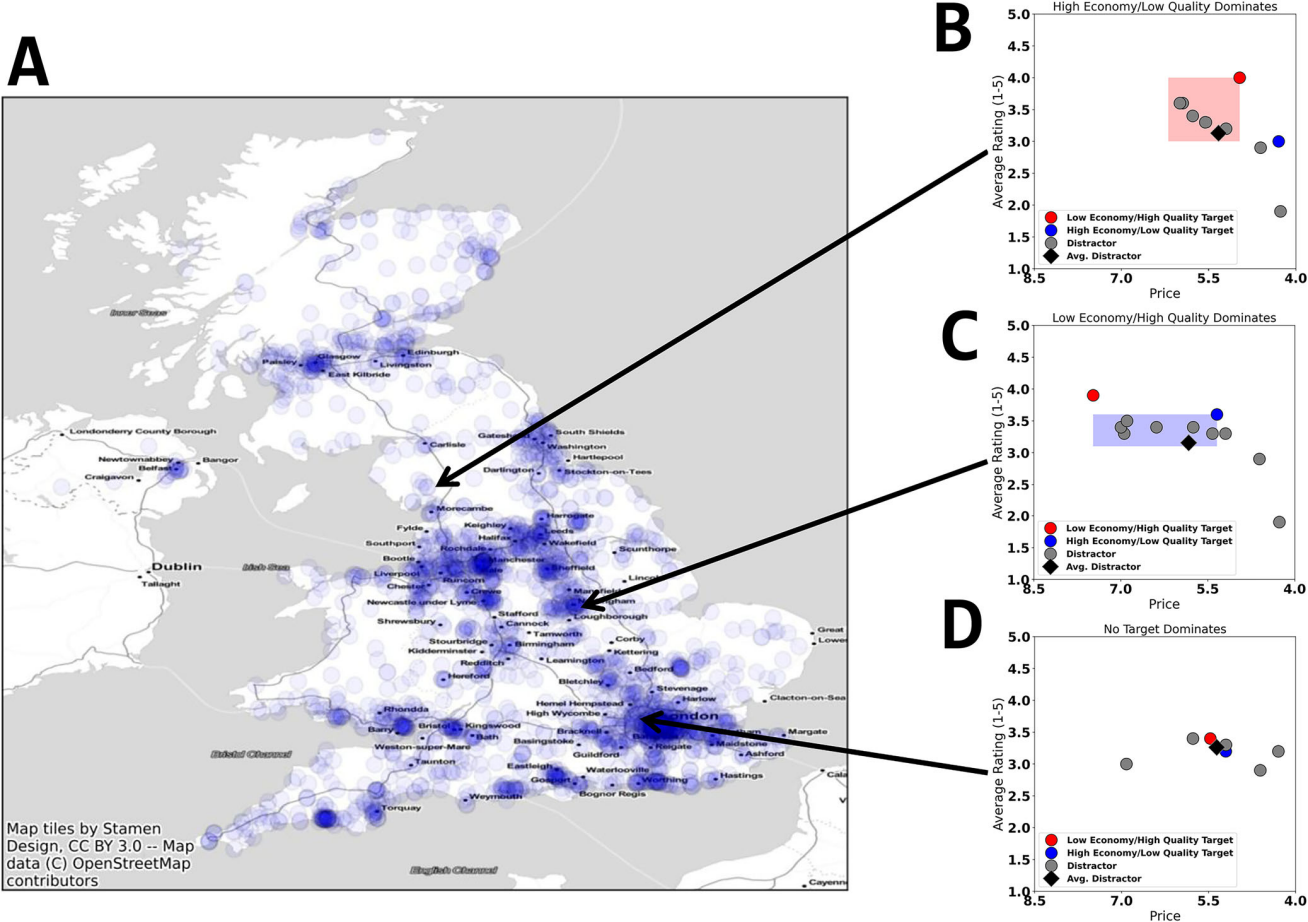
Illustration of example choice set construction. **A** Store locations in the UK. Blue points represent individual stores. Darker spots indicate areas with many store locations. **B** Example choice set where the higher quality, but more expensive, wine dominates. The red point represents the low economy, high quality target item, the blue point represents the high economy, low quality target item, and the grey points represent distractors. Targets are 1) popular and 2) equally preferred, on average, across sets. The red shaded area shows the “area of dominance”, in which the target item dominates over distractors (i.e., it is cheaper and higher quality than distractors, yet distractors are worse quality than the alternative target in red). The black diamond represents the average distractor, with coordinates equal to the mean of all distractor prices and ratings. **C** Example choice set where the less expensive, but lower quality wine dominate the set. The blue shaded area shows the “area of dominance”, in which the target item dominates over distractors (i.e., it is cheaper and higher quality than distractors, yet distractors are cheaper than the alternative target in red). The black diamond represents the average distractor, with coordinates equal to the mean of all distractor prices and ratings. **D** Example choice set where neither target wine dominates the set. Notice that the black diamond, the average distractor, is not dominated in either dimension: it is less expensive than the red target, but lower quality, and higher quality than the blue target, but more expensive.

Grocery transaction records, corresponding to purchases made using customer loyalty cards, were provided to us through a collaboration with a large a grocery store chain in the United Kingdom (see Fig. 2A). Each transaction record consists of a list of products, corresponding to a customer’s purchases on a visit to a particular store location. From these transaction records, we extracted all red and white wine purchases over a three-month period (225 unique red wines, 149 unique white wines), resulting in a total of roughly 11 M transaction across 1.2 M customers, from August to October 2019. For each product in a transaction, the following data were available: a unique store ID, a unique product ID, an anonymized customer ID (based on customer loyalty cards), the time and date at which the transaction took place, the sales price of the item at purchase, the quantity purchased. Additionally, a small proportion of transactions were marked as being on promotion or on discount, but as these purchases occurred infrequently in our dataset (5.6% of transactions), we excluded these records from our analyses, including the construction and analysis of choice sets described below.

Descriptive information about the full dataset is presented in Supplementary Table 1. To ensure that options effectively competed with each other, we excluded transactions for which more than one wine with the same product ID was purchased (20% of purchases, see Supplementary Fig. 1). Finally, we excluded transactions made in very small (or infrequently visited) stores, defined as the bottom 5th percentile of stores with respect to number of total transactions represented in the dataset, as these did not generate enough purchasing data to infer choice sets (described below). After applying these exclusions, our analyses examined 3.6 M wine purchases made by 755,158 unique customers (see Supplementary Table 1).

### Option attributes

Our analysis of decoy effects considered two attributes: *price* and *quality*. Item price (in GBP) was directly available in the dataset as the purchase price. Following past work^15^, our analyses consider the negative value of this attribute (-1 ⨉ price)—which we termed “economy,” whereby higher values of the economy attribute reflect lower prices.

Wine quality was estimated by computing the mean star rating of the item from Vivino (https://www.vivino.com), a popular website and smartphone app where users rate wines on a 1–5 star scale. We chose this proxy measure of wine quality for two reasons. First, by virtue of its large user base (29 M users), ratings are available for the vast majority of wines in our dataset. Second, past work finds that Vivino ratings reflect both consumers’ preferences and experts’ ratings, suggesting that these ratings are informative about ‘ground-truth’ wine quality, independently from price^42,43^. For each wine in the dataset, we computed a wine’s average rating of quality (in stars) from all user ratings provided for that wine. The median number of user ratings per wine analyzed was 448. Transactions pertaining to wines that did not appear in the Vivino ratings dataset were excluded from further analysis.

### Calculation of choice sets

We begin with a general description of our approach. To examine decoy effects in consumers’ wine choices, we first constructed choice sets which represented the plausible options that faced a consumer in store at the time of the purchase, guided by the following reasoning: if consumer A purchased wine X, consumer B purchased wine Y, and consumer Z purchased wine C at the same store on the same day, we infer that wines X, Y, and Z were part of the choice set that consumer A considered before choosing wine X. Thus, wines that are purchased on the same day at the same store together can be grouped across consumers, yielding inferred choice sets in which each observed wine purchase was presumably situated.

To do this, we took the following data processing steps. First, to ensure that the considered wines were adequately represented in the dataset, we constrained our analysis to include only wines that listed at least 1000 purchases across all available transaction records). In other words, we reasoned that infrequently purchased wines would complicate our choice set inference process as it is unlikely that multiple consumers would purchase the same unpopular wine at the same store on the same day. Next, to infer the choice set surrounding a particular wine purchase indicated in a transaction—that is, the other wine options that the consumer faced at the time of choice—we identified all other wines purchased at the same store on the same day by cross-referencing product IDs, consumer IDs, store IDs and dates of purchase.

Owing to the variability in store size (and consequently, selection of wines on offer), this approach resulted in choice sets of varying sizes. Our analyses was constrained to choice sets that contained between 5 and 20 wines (see Supplementary Fig. 2), which constituted the vast majority of total choice sets (85%), which resulted in 24,803 choice sets remaining in the final analysis (aggregated price and ratings of choice sets are visualized in Supplementary Fig. 3). We did this, chiefly, to ensure that the analyzed choice sets sizes were representative of choice set sizes typically encountered by consumers in our dataset. A consequence of this choice set inference approach is that very unpopular (i.e., infrequently purchased) wines appear sparsely in our transaction data. Thus, we note that these estimated choice sets represent a lower bound of the true sizes of the choice sets facing consumers, and accordingly, represent sets of viable options consumers were faced with, rather than a (necessarily) complete reconstruction of all possible options facing a consumer.

### Identification of target and decoy options

Target options were defined as pairs of wine that are popular (i.e., frequently purchased overall in the dataset) and equally preferred. Importantly, target options trade off along two attributes—here, economy and quality—such that one item is cheaper, but of lower quality and the other is more expensive, but of higher quality. In our dataset, we identified target pairs as the 20 most frequently purchased pairs of popular wines (across the entire dataset) that met the following criteria: (1) one wine’s economy was higher (i.e., average purchase price was lower) and had lower rated quality than the other, and (2) both wines were chosen, across choice sets, with roughly equal preference, such that the observed choice likelihoods were no less than 75% in favor of an option across choice sets. We did this to ensure that choice sets of interest had viable target options that with attributes that effectively traded off and were comparable with respect to overall popularity.

While our approach mirrors traditional trinary choice paradigms insofar as the identification of target options—two target alternatives which trade off in attribute values^3^— choice sets in the present analysis can contain multiple distractor (non-target) rather than a single distractor option. For simplicity, we took the average quality (rating) and economy of each set of distractor options as a summary measure of the distribution of distractor options’ attribute values (Fig. 2B–D; we return to theoretical questions surrounding the analysis of multiple distractors in the Discussion).

We then examined each inferred choice set in which these 20 previously identified target pairs appeared, categorizing each choice set according to whether (or not) one of the target options dominated the average of the distractor options, which yielded three possible scenarios: choice sets where the low economy/high quality wine dominates the average of the distractors (Fig. 2B), choice sets where the high economy/low quality wine dominates the average of the distractors (Fig. 2C), and choice sets where neither wine dominates the average of the distractors (i.e., with distractors being similarly priced and/or rated to targets; Fig. 2D).

The key dependent measure in our analysis of decoy effects is the relative preference between target wines, computed across these three classifications of inferred choice sets.

### Individual differences

In addition to examining aggregate preferences, we also examined whether individual differences in the frequency with which customers purchased wines influenced their sensitivity to the decoy effects. To do this, we categorized shoppers as Frequent (18,652 shoppers) versus Infrequent shoppers (31,526 shoppers) by performing a median split (to maintain roughly comparable sample sizes) upon the frequency with which their customer ID number appeared in the inferred choice sets described above. Shopper type (frequent versus infrequent) was subsequently included as a predictor variable (deviance-coded) in the choice model described below.

### Inferential statistics

We estimated three regression models to test our three key hypotheses. First, we used a linear regression to examine the effects of quality and economy upon wine popularity, defined as the proportion of purchases of that wine (irrespective of choice set), which we log-transformed as these popularity scores were substantially skewed. Second, to examine decoy effects (our main analyses of interest), we estimated a logistic regression model to predict relative preference between target wines (e.g., a high-economy/low-quality wine versus a low economy/high quality wine) across inferred choice sets. Following previous work^15^, this regression only included choices made to one of the two target options. This model predicted relative preference between target options as a function of: (1) dominance category (whether the average of the distractor options was dominated by high economy/low quality target, the low economy/high quality target, or neither, which was taken as the intercept), (2) set size, to test for potential effects of the number of options in the choice set, (3) maximum quality of the choice set and (4) maximum economy in the choice set, to control for the possibility that the best option with respect to either choice dimension influenced choice, (5) average quality and (6) average economy of the choice set, to control for set-wide context effects^2^, and 7) whether the purchase occurred on a weekend (Friday to Sunday, coded (1) or weekday (Monday to Thursday; coded 0), because alcohol sales are known to increase precipitously on weekends^44^. We examined the main effects of each these variables upon wine preference, as well as their interactions with dominance category, such that the full choice model followed the following specification:

$$\begin{array}{l}{\rm{Choos}}{{\rm{e}}}_{{\rm{A}}}\, \sim \,\left({\rm{Intercept}}\left[{\rm{Non}}-{\rm{Dominated}}\; {\rm{Set}}\right]\right.\\\left.\qquad\qquad\qquad\qquad+{\rm{Dominance}}\; {\rm{Category}}\left[{\rm{High}}\; {\rm{Econ;Low}}\; {\rm{Qual}}\right]\right.\\\left.\qquad\qquad\qquad\qquad+{\rm{Dominance}}\; {\rm{Category}}\left[{\rm{Low}}\; {\rm{Econ;High}}\;{\rm{Qual}}\right]\right)\,\\\qquad\qquad\qquad\qquad*\left({\rm{Set}}\;{\rm{Size}}* {\rm{Weekend}}\right)+{\rm{Max}}\left({\rm{Ratin}}{{\rm{g}}}_{{\rm{set}}}\right)+{\rm{Max}}\left({\rm{Pric}}{{\rm{e}}}_{{\rm{set}}}\right)\\\qquad\qquad\qquad\qquad+{\rm{Mean}}\left({\rm{Ratin}}{{\rm{g}}}_{{\rm{set}}}\right)+{\rm{Mean}}\left({\rm{Ratin}}{{\rm{g}}}_{{\rm{set}}}\right)\end{array}$$

(where + refers to the addition of a main effect and * refers to an interaction term and associated main effects). All continuous variables were centered using their median value.

Third, in follow-up analyses examining the proportion of dominated distractors, we estimated the same logistic regression model but replaced the “dominance category” predictor with continuous predictor variables representing the proportion of distractors dominated by the high-economy/low-quality target, and the proportion of distractors dominated by low the economy/high quality target (both of which were centered at their median value). All reported confidence intervals are at the 95% level. For each model we report its Cox-Snell Pseudo *R*^2^ value.

## Results

### Influence of product attributes on choice

**Fig. 3:**
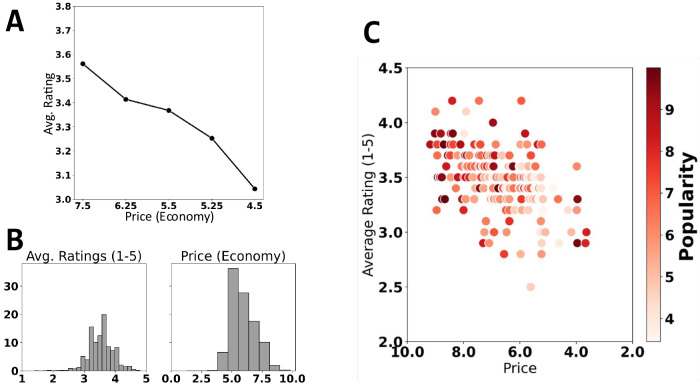
Influence of product attributes on choice. **A** Relationship between wines’ price and quality (star rating). The figure shows a strong negative relationship, suggesting that price and quality of wines are inversely related. **B** Histograms of ratings (left) and price (right). **C** Relationship between wines’ quality (star rating), economy (negative price), and popularity (standardized log number of purchases). The relationship between economy and quality is negative, but importantly both less expensive and higher quality wines independently predict more popular wines.

A precondition for examining decoy effects in multi-attribute choice is that the attributes effectively trade off—here, higher-quality wines should be more expensive and cheaper wines should, in general, be of lower quality. In validation of this, we observed a robust negative relationship between wine quality—computed from consumer ratings (see Methods)—and economy, such that that higher-rated wines were more expensive than lower rated wines (ratings predicting price: *b* = −1.76, *p* = < 0.0001, CI = [−1.76, −1.75]; R^2^ = 0.25; Fig. 3A). Furthermore, both attributes independently predicted overall rates of wine purchase: economy (b = 0.52, *p* < 0.0001, CI = [0.51, 0.52]) and rating (*b* = 0.35, *p* < 0.0001, CI = [0.34, 0.35]) significantly and positively predict wine purchase rates in our dataset, such that, controlling for quality, inexpensive wines are purchased more frequently, and, controlling for price, high quality wines were purchased more often (see Fig. 3B). Together, these results demonstrate that consumers’ wine choices were jointly informed by the quality and price attributes of the options, which effectively trade off with one another.

### Decoy effects

**Fig. 4:**
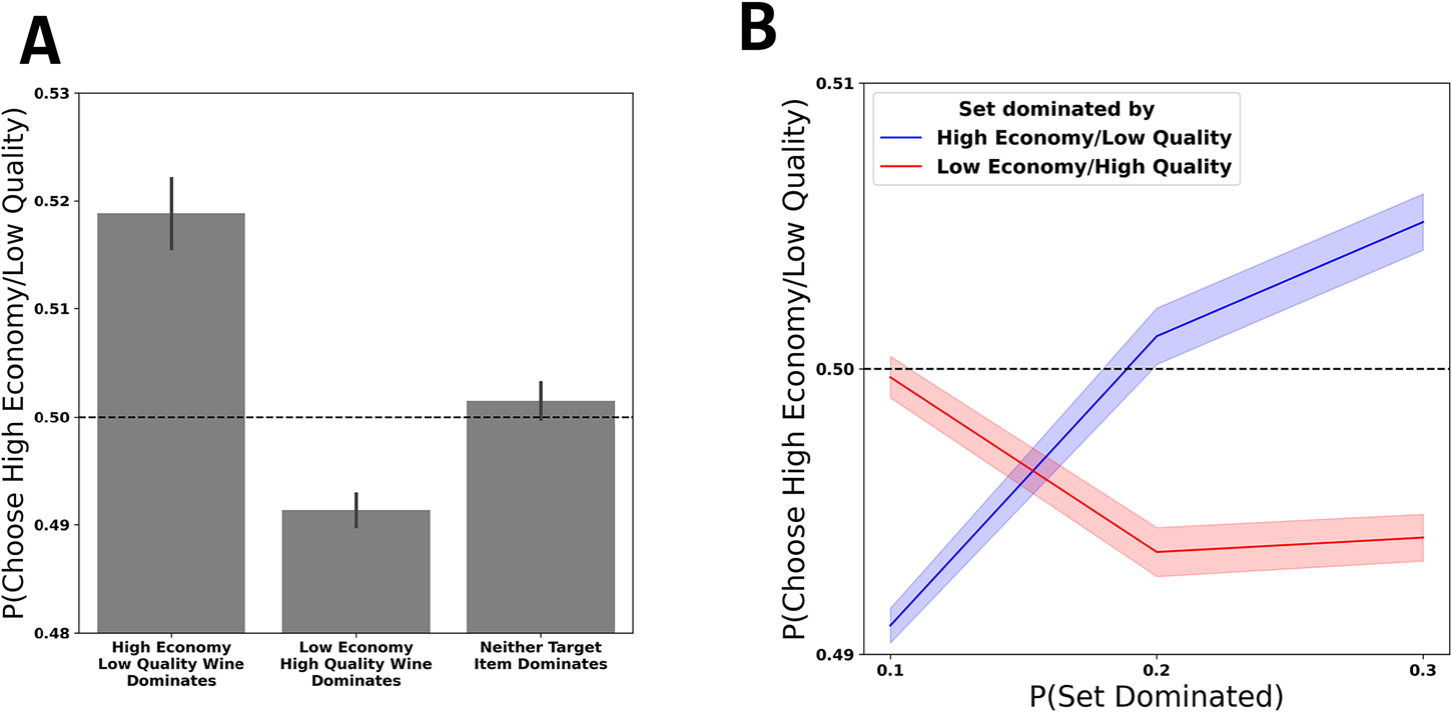
Attraction effects manifesting in constructed choice sets. **A** Decoy effects in inferred choice sets. The x-axis represents which target item dominates the set on average. The y-axis represents relative preference for one target over another, with higher values indicating a preference for high economy, low-quality, target wines and lower values indicating a preference for higher quality, but more expensive, wines. The dashed line represents indifferences. Error bars represent standard error of the mean. **B** Relative preference for targets as a function of relative set dominance. The x-axis here represents the proportion of distractors in a set that are dominated by a given target, depending on whether that target dominates the set overall (colour). In both cases, there is a linear relationship between the proportion of dominated distractors in a set and the relative preference between targets (y-axis). Bands represent standard error of the mean.

To examine decoy effects in consumers’ wine choices, we constructed choice sets which represented the plausible options that faced consumers in store at the time of the purchase (see Methods for details). Using these inferred choice sets, we investigated how choices between pairs of target wines—ubiquitous pairs of popular wines whose quality and economy attributes effectively trade off, and which are chosen roughly equiprobably in the aggregate—are influenced by the presence (versus absence) of decoy options dominated by one of the two target wines (see Fig. 2B–D for example situations). Fig. 4A depicts the observed preferences between target pairs in our dataset. In line with the traditional attraction effect (Huber et al. 1982), we observed that when neither target option dominates the other options in the choice set, consumers were indifferent between target options—that is, preference to purchase one target option over another was not significantly different from 50% (*b* = 0.002, *p* = 0.361, CI = [−0.003, 0.008]; see Supplementary Table 2 for full logistic regression coefficient estimates), which we also expected on the basis of our definition of target wine pairs.

However, when the distractor options in the choice set are dominated by a low-cost, low-quality target option, preference for the high-economy, low-quality target option over the opposing target pair increased significantly, beyond the point of indifference (*b* = 0.08, p < 0.0001, CI = [0.07, 0.09]). Similarly, when the distractor options were dominated by low-economy, high-quality target options, choices significantly favored low economy/high quality wines (*b* = −0.04, *p* < 0.0001, CI = [−0.05, −0.04]). Importantly, our logistic regression model controlled for a number of variables including the set size (i.e., number of options in the choice set; Supplementary Fig. 4) and day of the week (Supplementary Fig. 5).

These systematic choice biases are the signatures of the attraction effect, which is typically studied in the context of trinary choice, where a single distractor item influences choice between two target options (Dumbalska et al.). Here, our dataset contains choice sets that vary, both with respect to the number of distractor options, and the attribute values of distractors, in relation to the target items. That is, for a given target wine option, the proportion of distractor options dominated by that target option can differ considerably across choice sets (Supplementary Fig. 6), permitting us to investigate whether the proportion of items dominated by a target wine option modulates the strength of the observed attraction effect (Fig. 4B). Interestingly, we observed a linear relationship between the proportion of distractor options dominated by a target wine and the strength of preference for that wine over the corresponding target option (high economy/low quality: *b* = 0.11, *p* < 0.0001, CI = [0.08, 0.14]; low economy/high quality: *b* = −0.03, *p* = 0.008, CI = [−0.05, −0.01]; see Supplementary Table 3 for full output). In other words, in choice sets where a target dominated a larger share of distractor options, we observed stronger bias towards the dominating target wine. Again, this effect was robust when controlling for set sizes (Supplementary Fig. 7) and purchase day (Supplementary Table 3).

### Individual differences in frequency of shopping

The above analyses suggest that, in the aggregate, the attraction effect manifests in the sorts of consumer choices examined here. However, unlike traditional lab experiments, where options are often unfamiliar to participants, here consumers’ real-world purchasing decisions are informed by unique histories of past choices —and consumption experience—with the options they face^29^. For instance, a frequent shopper may rely less on described information about a product’s attributes, instead relying on their past experience with those options—akin to a habitual decision style^45^. Thus we might expect experienced consumers, with strongly developed preferences, would be less susceptible to decoy effects^46,47^.

**Fig. 5:**
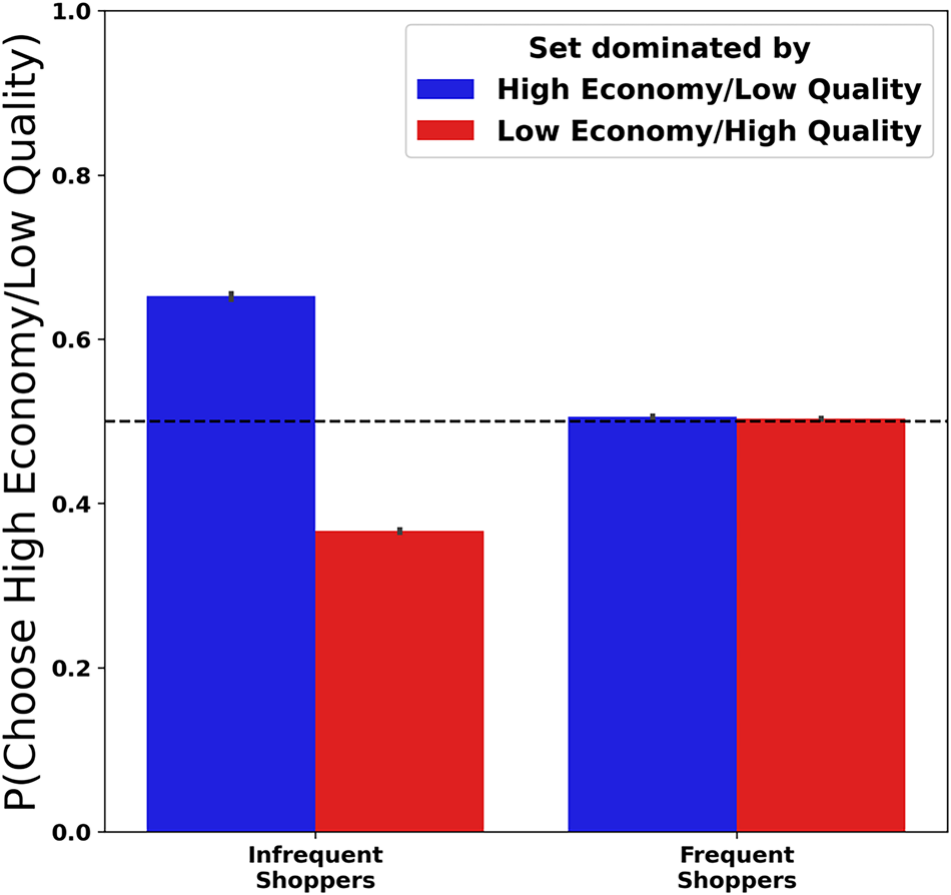
Decoy effects in constructed choice sets plotted separately for frequent and infrequent shoppers (as defined by median split of wine purchasing frequency). The x-axis represents shopper type on the basis of wine buying frequency. The colour represents which target item dominates the set on average. The y-axis represents relative preference for one target over another, with higher values indicating a preference for high economy, low-quality, target wines and lower values indicating a preference for higher quality, but more expensive, wines. The dashed line represents indifferences. Error bars represent standard error of the mean.

To test this question, we grouped shoppers in our dataset according to their wine purchasing frequency (via median split; see Methods for details), analyzing target option preferences as in the aggregate analyses above. As illustrated in Fig. 5, we observed that the attraction effect was strongly attenuated for shoppers who purchased wine more frequently, as indexed by a significant interaction between shopper type and distractor dominance. Namely, preferences between high-economy/low-quality targets (*b* = −1.09, *p* < 0.0001, CI = [−1.12, −1.05]) and low-economy/high-quality target (*b* = 0.092, *p* < 0.0001, CI = [0.07, 0.11]) approach indifference for frequent wine buyers, while infrequent wine buyers exhibited a marked attraction effect in line with the aggregate results depicted in Fig. 4.

## Discussion

The present study examined whether the attraction effect, a canonical decision-making bias and a hallmark of context-dependent choice—manifest in a large dataset of wine purchases in the UK, Specifically, we found that wine purchases were systematically biased in favor of wines that dominated choice sets—that is, in favor of wines that were both higher quality and less expensive than distractor wines in a choice set. Additionally, we found that the strength of this bias increased monotonically according to the relative degree of dominance that the target exerted over distractors (Fig. 4B), such we observed stronger preference for target options in choice sets where the target dominated a larger share of decoy options. Finally, we found that sensitivity to this effect was modulated by consumers’ history of experience with products—indexed here by their frequency of wine purchases—such that consumers who bought wines frequently exhibited far less biased choice than those who bought relatively fewer wines^46,47^. Taken together, these results corroborate and extend a large body of work which suggests that economic decisions—both in the lab^3,7,8,15^ and externally^2,28^—are systematically biased by the presence of irrelevant alternatives, violating the “independence of irrelevant alternatives” axiom of rational choice^48,49^.

Furthermore, by leveraging a large and diverse dataset of real-world purchases, we were able to look beyond traditional trinary (three-option) paradigms and examine decoy effects in more realistic consumer choice settings, in which multiple distractor options are often present in a choice set. To our knowledge, our study provides the first systematic examination of the influence of *distributions* of decoy options upon choice. Doing so allowed us to uniquely probe how choice context composition—here, the number of dominated options—influenced sensitivity to the attraction effect. Using the average value of distractor items across attribute dimensions, we find not only aggregate evidence for decoy effects in large-scale purchasing behavior, but also observe that the strength of this decoy effect is modulated by the number of dominated options in a choice set. Theoretically, the observation of systematic attraction effects in choice sets with multiple decoy options highlights the potential generalizability of this effect to more complex choice settings outside the laboratory. Practically, this observation suggests that retailers and platform designers should consider the possibility that consumers—to the extent they identify options as dominated—may be influenced systemically by multiple decoy options.

For the sake of simplicity—and owing to limitations in the present dataset—we used the average attribute values of the distractor items to characterize the ‘center of mass’ of possible influence of distractor items. While we found that the position of the average distractor item with respect to the target options systematically influenced preferences, giving rise to an attraction-like effect, it is worth noting the multitude of descriptive statistics that could be used to characterize the geometric configuration of distractor items. Indeed, it is possible that the average itself may be overly simplistic given the complexity of the decision process under examination. For instance, laboratory studies—again, examining trinary choice—have found that the distance between a distractor and its dominating target option bears influence on the strength of observed attraction effects^15,18^. While the present study provides an important proof-of-concept demonstrating that traditional decoy effects are observable in the more realistic settings with multiple distractor items, future work should examine, more systematically, how the geometry of distractor item distributions influences target preferences, both in the laboratory and in real-world purchasing behavior. These findings, in turn, might hold important implications for extending models of context effects^8,26,50,51^ to settings beyond trinary choice.

It is also worth noting the modest effect size we observed in our analysis. The immense dataset analyzed in our approach uncovered decoy effects with small magnitudes but very narrow confidence intervals, suggesting that, in the long run, the magnitude of these effects are particularly constrained (albeit significantly greater than zero). Underlining this point, scholars have advocated for increasing emphasis on reporting magnitudes of effects, and decreasing emphasis on the statistical significance status of effects^52,53^. The subtle effect we observe in this naturalistic setting is perhaps unsurprising given the modest effect sizes observed in recent^54^ examinations of the attraction effect in well-controlled, laboratory-based settings^15,22,32,51^ and the heterogeneity of observed decoy effects in field experiments^24^.

Nonetheless, these results demonstrate that our analysis approach is capable of detecting attraction-like effects in an uncontrolled naturalistic setting with multiple, competing sources of choice variability. At the same time, taking an economic perspective, a 1–2% shift in consumer preferences towards more expensive wines could amount to an increase in revenue of roughly £19,000 for the choice sets analyzed over the three months examined, which could be advantageous for firms given the presumably low cost of facilitating an attraction effect—for example, by re-arranging choice sets to increase the salience of dominated distractor wines.

Finally, it is important to note that the choice sets uncovered by our analysis approach should be taken as estimates of the “ground-truth” choice sets shoppers actually faced by shoppers. As a result of our choice set inference approach, it is possible that disparities between inferred versus ground-truth choice sets could arise from infrequently-purchased distractor wines (owing to their sparse representation in daily purchase records) or shelf re-stocking in the course of a business day (which is not represented in purchase records). These disparities could engender potential issues for examining decoy effects for several reasons. First, our approach could omit non-viable decoy options which are completely dominated by both target options, and consequently, are absent from transaction records as they are never chosen. From a theoretical perspective, these options would constitute the “purest” decoys (because they are genuinely undesirable), but for practical purpose stores will not stock such items for long periods of time. Second, and relatedly, it is possible that shoppers do not perceive, visually or otherwise, a given domination relationship when making their choice. We aimed to mitigate this possibility by constructing choice sets from transaction records that are both temporally (the same days) and geographically proximate (the same stores), but these choice sets, again, represent an approximation of the “true” choice set faced by consumers.

Additionally, as highlighted by our individual differences analysis and past work^38^, highly experienced wine consumers may differ from less experienced consumers with respect to the information they use to make wine choices. In the present study, we found that an individual’s level of wine purchasing experience manifests in their susceptibility to decoy effects, such that more frequent wine buyers exhibit smaller (or non-existent) decoy effects.

It is possible that frequent shoppers may be better acquainted with the quality of individual wines, possibly owing to these consumers’ reliance on extensive personal experiences with these wines—rather than external information and/or use external tools (e.g., online reviews, social media) to inform their decisions. For these more frequent shoppers, our use of wine ratings may not be as accurate a reflection of perceived wine quality. Nonetheless, we believe that the selectively of the observed decoy effect to consumers with less extensive experience purchasing wine provides an informative demonstration of potential boundary conditions on decoy effects as well as elucidates the heterogeneity decision-makers’ susceptibility to decoy effects.

The finding that frequent wine buyers exhibit diminished—or even non-existent—decoy effects is consistent with recent work proposing that frequent consumers may rely on habitual consumption strategies, rather than strategies that aim to maximize computed utility^45^. From this perspective, the influence of dominated distractor items on choice for consumers with deeply engrained purchasing habits—which, again, is difficult to operationalize in the context of laboratory studies of decision-making^55^— nonetheless warrants further inquiry.

## Supplementary information

Supplementary information

## Data Availability

The dataset used in this study is commercially sensitive and subject to strict access controls. To request route to access, please contact the Neodemographics Laboratory (N/Lab) director, James Goulding (james.goulding@nottingham.ac.uk). We will promptly provide guidance on engaging with the commercial retailer and endeavour to respond within one month.

## Code availability

The code used in this study is commercially sensitive and subject to strict access controls. To request route to access, please contact the Neodemographics Laboratory (N/Lab) director, James Goulding (james.goulding@nottingham.ac.uk). We will promptly provide guidance on engaging with the commercial retailer and endeavor to respond within one month.
