# Publisher Correction: How decoy options ferment choice biases in real-world consumer decision-making

**DOI:** 10.1038/s41539-025-00387-2

**Published:** 2025-12-16

**Authors:** Sean Devine, James Goulding, John Harvey, Anya Skatova, A. Ross Otto

**Affiliations:** 1 Department of Psychology, McGill University, Montreal, Canada; 2 N/LAB, Nottingham University Business School, Nottingham, UK; 3 Digital Footprints Lab & Medical Research Council Integrative Epidemiology Unit at the University of Bristol, Population Health Science, Bristol Medical School, University of Bristol, Bristol, UK

**Keywords:** Human behaviour, Decision making

Correction to: *npj Science of Learning* 10.1038/s41539-025-00341-2, published online 22 August 2025

In this article, the Figure legends were incorrectly assigned. The original article has now been corrected.

