# Supplementary material for: How decoy options ferment choice biases in real-world consumer decision-making
Source: NPJ Sci Learn. 2025 Aug 22;10:60. doi: 10.1038/s41539-025-00341-2 (PMC12371001; doi:10.1038/s41539-025-00341-2)
Supplement: Supplementary file 1 — Supplementary information [file 41539_2025_341_MOESM1_ESM.pdf]

Supplemental Materials for:
How Decoy Options Ferment Choice Biases in Real-World Consumer Decision-Making

Sean Devine*[1], James Goulding[2], John Harvey[2], Anya Skatova[†3] & A. Ross Otto[†1]

[1] Department of Psychology, McGill University, Montreal, Canada
[2] N/LAB, Nottingham University Business School, United Kingdom
[3] Bristol Medical School, Bristol University, Bristol, United Kingdom

Corresponding author: seandamiandevine@gmail.com
† These authors contributed equally

Author contributions: S.D. analysed and visualized the data and co-authored the manuscript. A.S. co-supervised the project and co-authored the manuscript. J.G. co-supervised the project, procured the raw dataset, and co-authored the manuscript. J.H. procured and curated the raw dataset and co-authored the manuscript, A.R.O. supervised the project, secured funding, and co-authored the manuscript.

Competing interests: The authors declare no conflict of interest.

Data availability: The dataset used in this study is commercially sensitive and subject to strict access controls. To request route to access, please contact the Neodemographics Laboratory (N/Lab) director, James Goulding (james.goulding@nottingham.ac.uk). We will promptly provide guidance on engaging with the commercial retailer and endeavour to respond within one month.

Code availability: The code used in this study is commercially sensitive and subject to strict access controls. To request route to access, please contact the Neodemographics Laboratory (N/Lab) director, James Goulding (james.goulding@nottingham.ac.uk). We will promptly provide guidance on engaging with the commercial retailer and endeavour to respond within one month.

Acknowledgements: This work was supported by a grant (awarded to ARO), a from NSERC fellowship (awarded to SD) and a UKRI (MR/T043520/1) Future Leaders Fellowship to AS.

Supplemental Figures

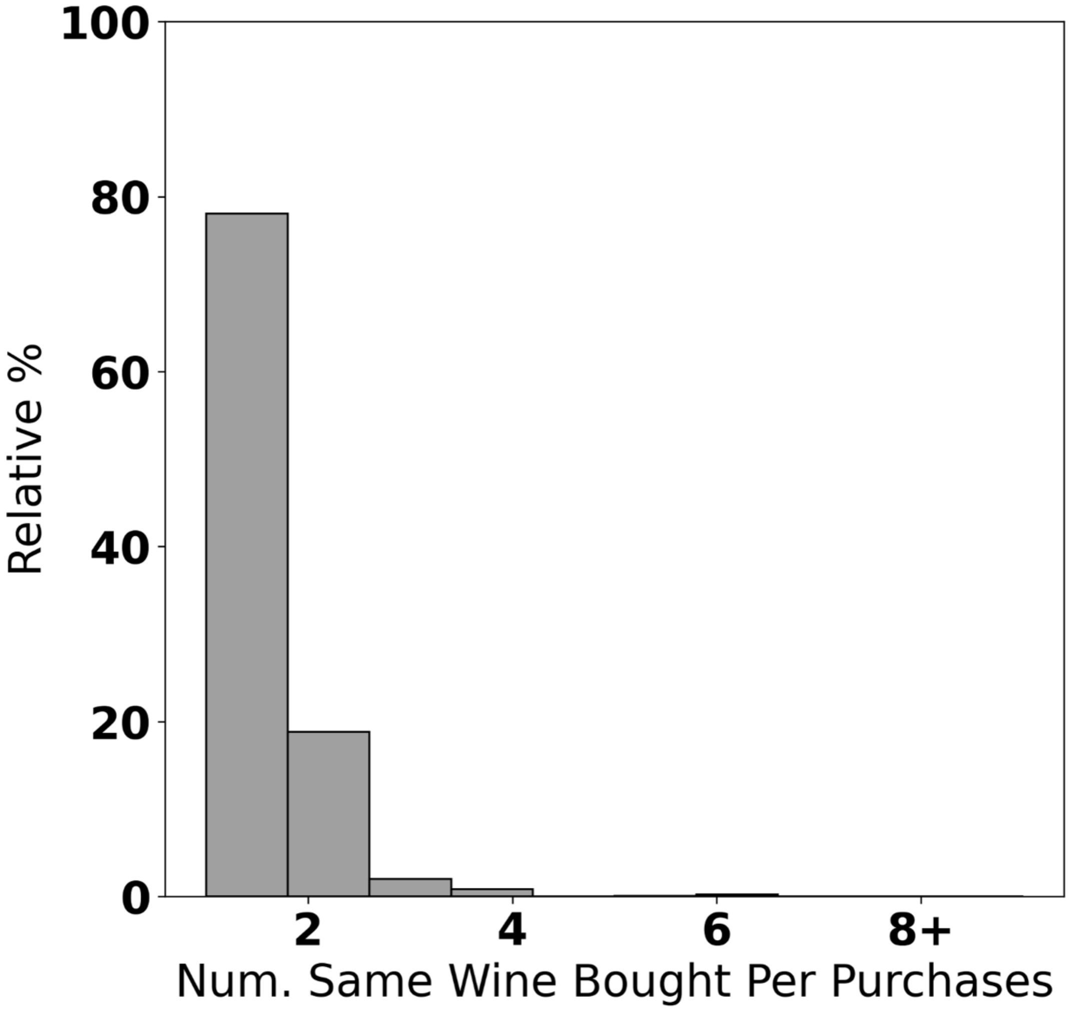

Supplementary Figure 1. Number of sales for the same wine in the same transaction. In the full analysis, only the case of one wine per purchase was considered.

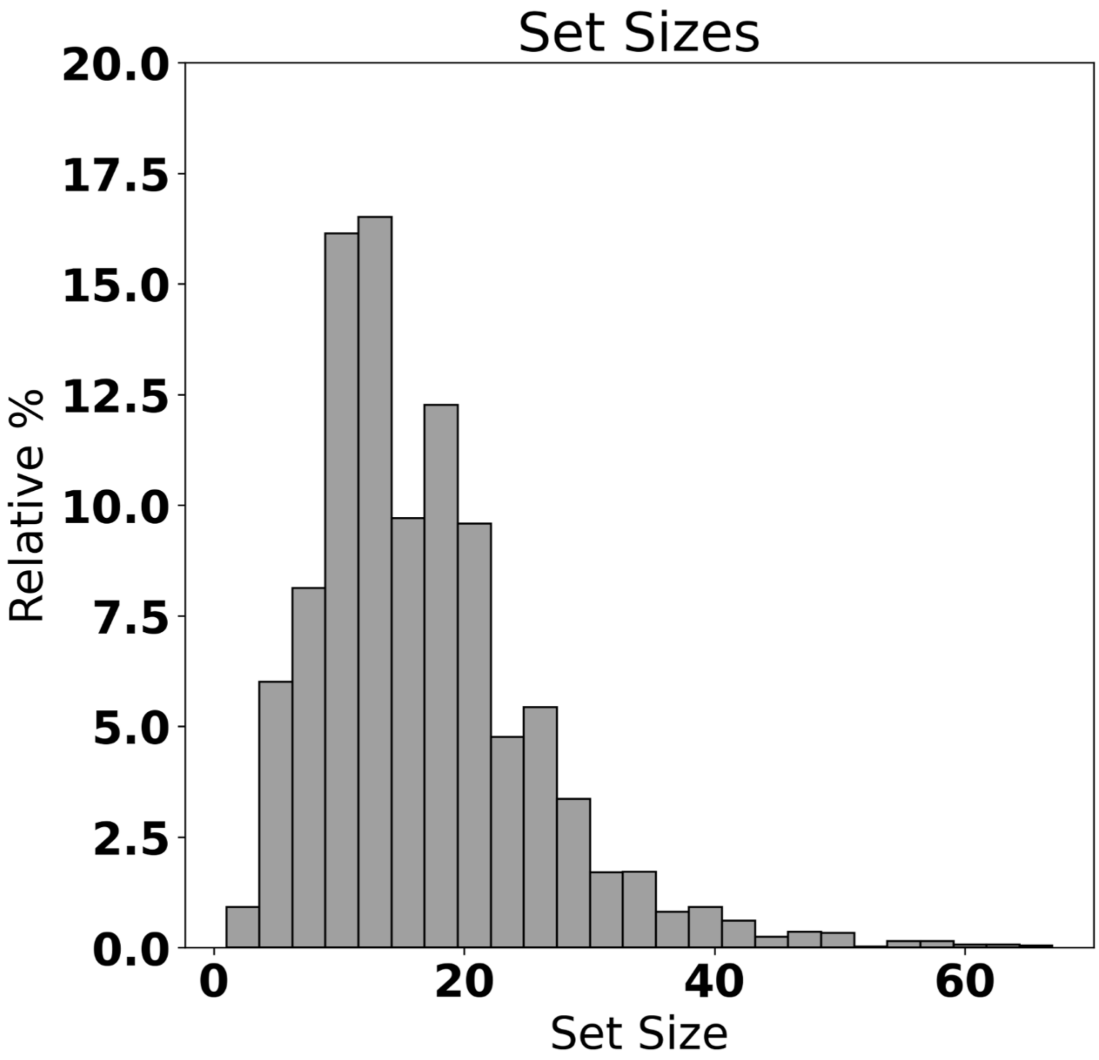

Supplementary Figure 2. Distribution of set sizes from constructed choice sets. As described in the main text, choice set sizes with very few observations were excluded from further analysis, such that the main analysis focused on sets sizes 5 and 20.

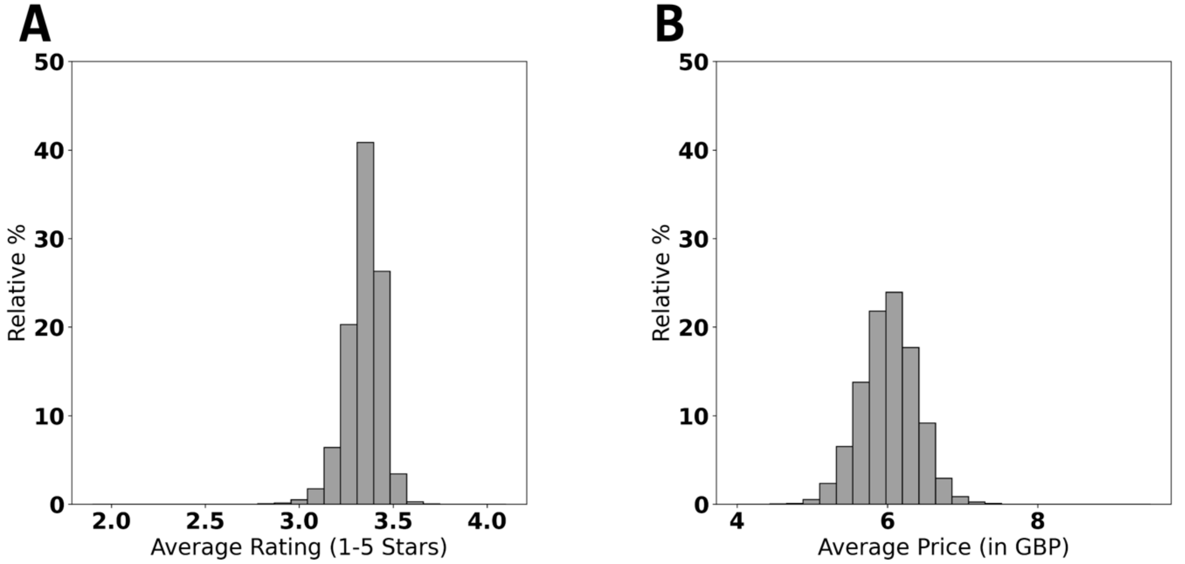

Supplementary Figure 3. Aggregated average (A) star ratings and (B) price across constructed choice sets.

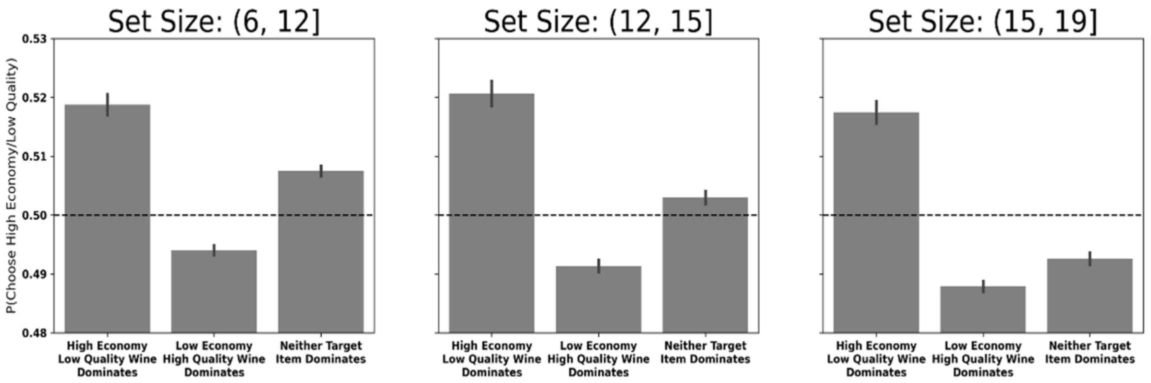

Supplementary Figure 4. Decoy effects in constructed choice sets across choice set size. The x-axis represents which target item dominates the set on average. The y-axis represents relative preference for one target over another, with higher values indicating a preference for high economy, low-quality, target wines and lower values indicating a preference for higher quality, but more expensive, wines. The dashed line represents indifferences. Errorbars represents standard error of the mean. Panels represent choice sets of different sizes. As can be seen, across choice set size, the pattern is the same as in the aggregated analysis presented in the main text (Figure 4A).

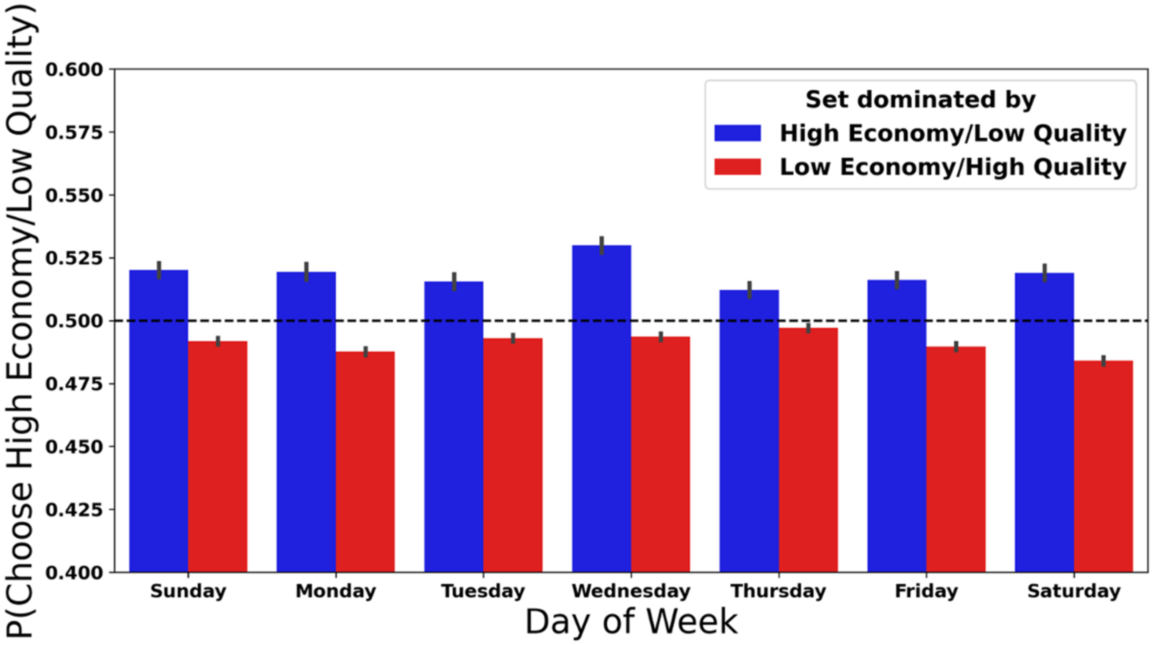

Supplementary Figure 5. Decoy effects in constructed choice sets across days of the week in which purchases were made. The x-axis represents the day of the week. Colours represent which target item dominates the set on average. The y-axis represents relative preference for one target over another, with higher values indicating a preference for high economy, low-quality, target wines and lower values indicating a preference for higher quality, but more expensive, wines. The dashed line represents indifferences. Errorbars represents standard error of the mean. As can be seen, across days, the pattern is the same as in the aggregated analysis presented in the main text (Figure 4A).

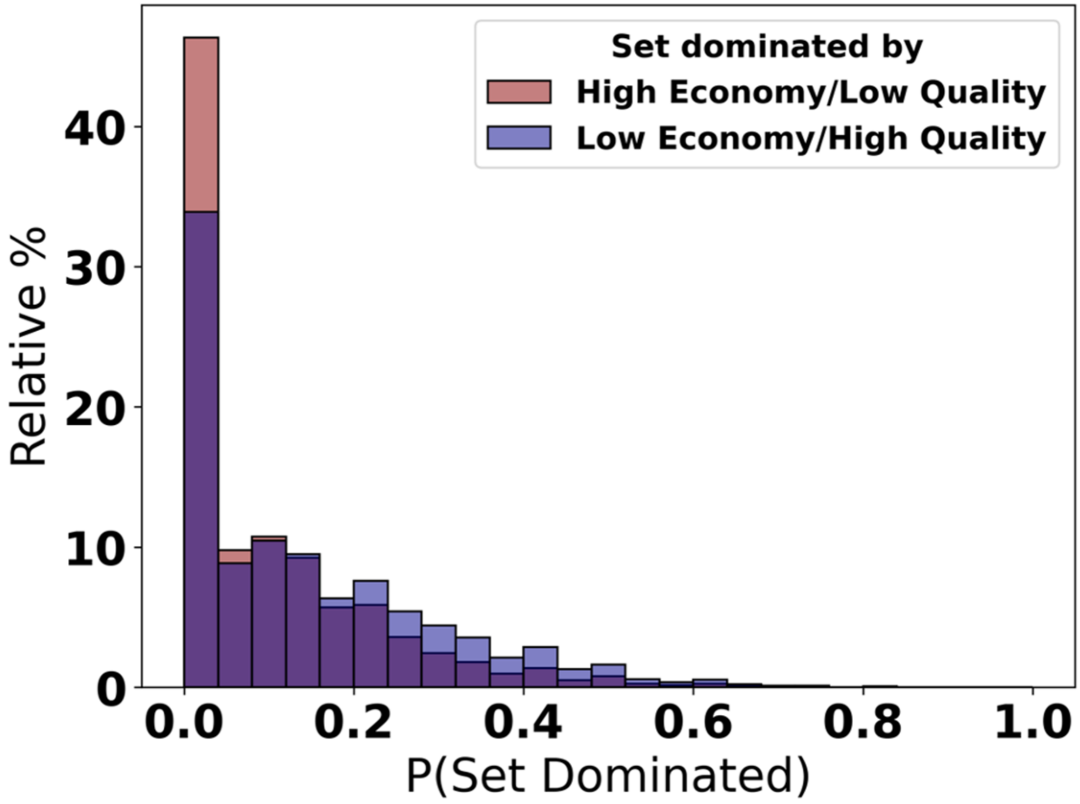

Supplementary Figure 6. Distribution of the number of distractors in a choice set that were dominated by a given target, either an inexpensive, but low quality, target (blue) or an expensive but high quality target (red)

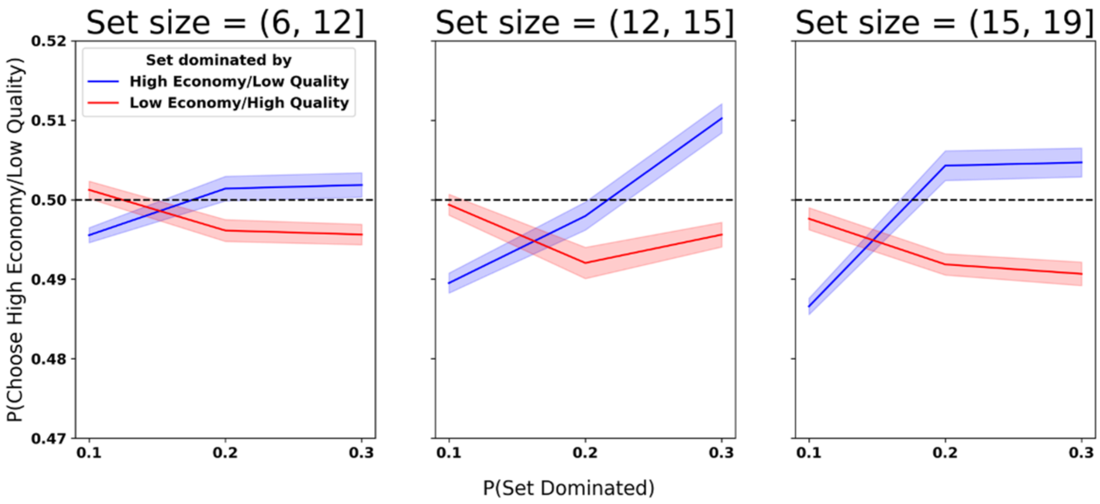

Supplementary Figure 7. Relative preference for targets as a function of relative set dominance across set sizes. The x-axis here represents the proportion of distractors in a set that are dominated by a given target, depending on whether that target dominates the set overall (colour). In both cases, across set sizes, there is a linear relationship between the proportion of dominated distractors in a set and the relative preference between targets (y-axis). Titles indicate set size ranges (binned). Bands represent standard error of the mean.

Supplemental Tables

Supplementary Table 1. Sample description, pre- and post-exclusion.

	Pre-Exclusion	Post-Exclusion
Transactions	11 097 773	3 649 027
Customers	1 242 268	755 158
Stores	2673	2469
Wines	568	374

Supplementary Table 2. Coefficient estimates for logistic regression predicting target choice (high economy/low quality item) from dominance category and covariates. Note: High (Low) Econ./Low (High) Qual. refers to the set dominance category, such that the *b* columns of the High Econ./Low Qual. row refers the log odds of choosing the target in a set where the dominant wine is high in economy and low in quality. Pseudo-R2 = 0.003

	b	SE	P	CI 2.5%	CI 97.5%
Intercept [Non-Dominated Set]	0.0024	0.003	0.361	-0.003	0.008
Dominance Category [High Econ; Low Qual]	0.0836	0.006	<.0001	0.073	0.094
Dominance Category [Low Econ; High Qual]	-0.0417	0.003	<.0001	-0.048	-0.035
Set Size	-0.0059	0.001	<.0001	-0.007	-0.004
Dominance Category [High Econ;Low Qual] × Set Size	0.0048	0.002	0.002	0.002	0.008
Dominance Category [Low Econ;High Qual] × Set Size	0.0042	0.001	<.0001	0.002	0.006
Weekend	-0.0091	0.005	0.068	-0.019	0.001
Dominance Category [High Econ;Low Qual] × Weekend	0.0155	0.011	0.154	-0.006	0.037
Dominance Category [Low Econ;High Qual] × Weekend	-0.0059	0.007	0.384	-0.019	0.007
Set Size × Weekend	0.0008	0.001	0.556	-0.002	0.004
Dominance Category [High Econ;Low Qual] × Set Size × Weekend	-0.0021	0.003	0.481	-0.008	0.004
Dominance Category [Low Econ;High Qual] × Set Size × Weekend	-0.0009	0.002	0.647	-0.005	0.003
Max(Rating)	-0.0066	0.009	0.459	-0.024	0.011
Max(Price)	-0.0007	0.003	0.789	-0.006	0.005
Mean(Rating)	-0.1922	0.02	<.0001	-0.232	-0.152
Mean(Price)	0.0006	0.007	0.93	-0.014	0.015

Supplementary Table 3. Coefficient estimates for logistic regression predicting target choice (high economy/low quality item) from dominance category and covariates. Note: P(High (Low) Econ.; Low (High) Qual.) refers to the proportion to which a set is dominated by a given target, such that the *b* columns of the P(High Econ.; Low Qual.) row refers the log odds of choosing the target in a set for a 1-unit increase in the proportion of the set which is dominated by high economy/low quality targets. Pseudo-R2 = 0.004.

	b	SE	P	CI 2.5%	CI 97.5%
Intercept [Non-Dominated Set]	-0.018	0.002	<.0001	-0.022	-0.014
P(High Econ; Low Qual)	0.108	0.015	<.0001	0.08	0.137
P(Low Econ; High Qual)	-0.0314	0.012	0.008	-0.055	-0.008
Set Size	-0.0057	0.001	<.0001	-0.007	-0.005
P(High Econ./Low Qual.) × Set Size	0.0244	0.004	<.0001	0.017	0.032
P(Low Econ./High Qual.) × Set Size	0.0038	0.003	0.224	-0.002	0.01
Weekend	-0.0184	0.004	<.0001	-0.026	-0.011
P(High Econ./Low Qual.) × Weekend	0.1092	0.029	<.0001	0.053	0.166
P(Low Econ./High Qual.) × Weekend	-0.0035	0.023	0.88	-0.049	0.042
Set Size × Weekend	0.0002	0.001	0.821	-0.002	0.002
P(High Econ./Low Qual.) × Set Size × Weekend	-0.0048	0.008	0.541	-0.02	0.011
P(Low Econ./High Qual.) × Set Size × Weekend	0.0069	0.006	0.276	-0.005	0.019
Max(Rating)	0.0101	0.009	0.256	-0.007	0.027
Max(Price)	-0.0035	0.003	0.202	-0.009	0.002
Mean(Rating)	-0.1693	0.02	<.0001	-0.209	-0.13
Mean(Price)	0.0111	0.007	0.132	-0.003	0.02
